# Low percentage of surgeons meet the minimum recommended unicompartmental knee arthroplasty usage thresholds: Analysis of 3037 Surgeons from Three National Joint Registries

**DOI:** 10.1007/s00167-021-06437-7

**Published:** 2021-02-17

**Authors:** Antonio Klasan, David A. Parker, Peter L. Lewis, Simon W. Young

**Affiliations:** 1 Department for Orthopaedics and Traumatology, Kepler University Hospital GmbH, Krankenhausstrasse 9, 4020 Linz, Austria; 2 Johannes Kepler University Linz, Altenberger Strasse 69, 4040 Linz, Austria; 3 Sydney Orthopaedic Research Institute, Sydney, Australia; 4 Australian Orthopaedic Association National Joint Replacement Registry, Adelaide, Australia; 5 Wakefield Orthopaedic Clinic, Adelaide, Australia; 6 Faculty of Medicine, Department of Orthopedics, Lund University, Clinical Sciences Lund, Lund, Sweden; 7 North Shore Hospital, Auckland, New Zealand

**Keywords:** Total knee arthroplasty, Unicompartmental knee arthroplasty, Usage, Revision, Registry

## Abstract

**Purpose:**

The reported usage of UKA is around 10% in the UK, Australian and New Zealand joint registries. However, some authors recommend that a higher UKA usage of 20%, or a minimum 12 UKA cases per year, would reduce revision rates. The purpose of this study was to analyze the percentage of surgeons performing the recommended thresholds in these 3 registries.

**Methods:**

Data from the UK, Australian and New Zealand registry databases was utilized from the time period since their respective introduction until 2017. All primary TKA and UKA performed for the diagnosis of osteoarthritis by surgeons with more than 100 recorded knee arthroplasties in their respective registry were included. The results between the registries were compared and a pooled analysis was performed. The number of surgeons meeting the recommended caseload of > 20% UKA yearly or 12 UKA cases yearly was calculated.

**Results:**

We identified 3037 knee surgeons performing 1,556,440 knee arthroplasties, of which 131,575 were UKA (8.45%). Over 50% of knee surgeons in each registry had a proportion of less than 5% UKA of their knee replacement procedures. After pooling of data, median surgeon UKA usage was 2.0% (IQR 0–9.1%).

The percentage of surgeons meeting the proposed caseload criteria was highest in New Zealand, 16.3%, followed by the UK at 12.4% and Australia 11.3% (*p* = 0.28).

**Conclusion:**

More than 50% of knee surgeons in UK, Australian and New Zealand joint registries perform less than 5% of UKA yearly. The majority of experienced knee surgeons are not meeting the recommended minimum thresholds, which might indicate that the recommended thresholds are not feasible for the vast majority of knee surgeons. The reasons behind this require further research.

**Level of Evidence:**

Level III retrospective registry study.

## Introduction

Advanced medial knee osteoarthritis (OA) requiring an arthroplasty can be managed with a total knee arthroplasty (TKA) or a unicompartmental knee arthroplasty (UKA) [31]. A recent large systematic review and meta-analysis demonstrated better functional scores for UKA and no difference in pain [31]. Major complications and mortality were higher after TKA. However, the revision rate of UKA was reported to be 2.5–6 times higher [31]. These differences are a topic of considerable debate, with some arguing that fewer UKAs should be performed to reduce this revision risk, and others argue that the revision rate can be reduced by performing more UKAs [22]. Increasing the number of UKA is supported by reports suggesting that up to 48% of knee arthroplasty patients are suitable for UKA [30], and by a registry study from the United Kingdom reporting an acceptable revision rate when surgeons have at least 20% of UKA usage out of their complete knee arthroplasty caseload with optimal results between 40 and 60% UKA usage within their complete knee arthroplasty caseload [15]. Surgeons with a < 5% UKA caseload have the highest revision rate [15]. Alternatively, in another study a minimum yearly caseload of 12 UKA significantly decreased the revision rate and this is also recommended as a threshold [23].

The reported usage of UKA in the most recent New Zealand, Australian and UK joint registry reports are 11.5%, 8.5%, and 10.1% respectively [24–26]. These numbers incorporate all cases, including those performed by orthopedic generalists and therefore may not represent the practice of specialist knee surgeons.

The aim of this study was to determine UKA usage across three national registries, and to identify the of percentage of knee surgeons in each registry that meet the proposed minimum recommendations of 20% of UKA or 12 UKA cases yearly. We also aimed to analyze the trends in UKA usage over the duration of the study. Based on the overall UKA usage, we hypothesized that a low number of knee surgeons meet the recommended thresholds.

## Methods

Data was obtained from 3 national joint registries; National Joint Registry of England, Wales, Northern Ireland, the Isle of Man and States of Guernsey (NJR), Australian Orthopaedic Association National Joint Registry (AOANJRR) and the New Zealand Joint Registry (NZJR). The registries provided data for primary TKA and UKA procedures, stratified by surgeon ID, for each year, from the commencement of the registry until 31 December 2017. The UK and Australian registries each covered 15 years, and the New Zealand registry 18 years. Both UKA and TKR arthroplasty procedures were included, while revision knee procedures, patellofemoral replacement, bicompartmental and partial resurfacing knee procedures were excluded. We also limited the study to include only surgeons who had a minimum of 100 arthroplasty procedures recorded within their respective national joint registry. The number of surgeons meeting the recommended threshold of > 20% UKA or 12 UKA procedures/year [23] was analyzed for each registry.

### Statistical analysis

Cumulative numbers and frequencies were reported for each registry. Yearly UKA usage by caseload was calculated by dividing the number of UKA performed with the number of years of activity in the registry. Yearly UKA usage percentage was calculated as the percentage of UKA in all KA for each surgeon. For non-normally distributed data, median and range were reported in addition to the mean. A two-step cluster analysis was performed to determine the clusters. With the distribution of UKA percentages, and with one cluster set to a minimum of 20% UKA [15], the hierarchical cluster analysis determined 6 clusters: < 1%; 1–5%; 5–10%; 10–20%; 20–30% and > 30%. Correlation between total procedure numbers and UKA usage was assessed using the Pearson correlation. A two-step cluster analysis was used to determine the number of usage clusters and the usage percentages for each cluster. After the between registry comparison was performed, the data was pooled for further analysis. Statistical significance was set at *p* < 0.05. SPSS 24.0 (IBM, Armonk, NY, US) was used for statistical analysis.

## Results

**Fig. 1:**
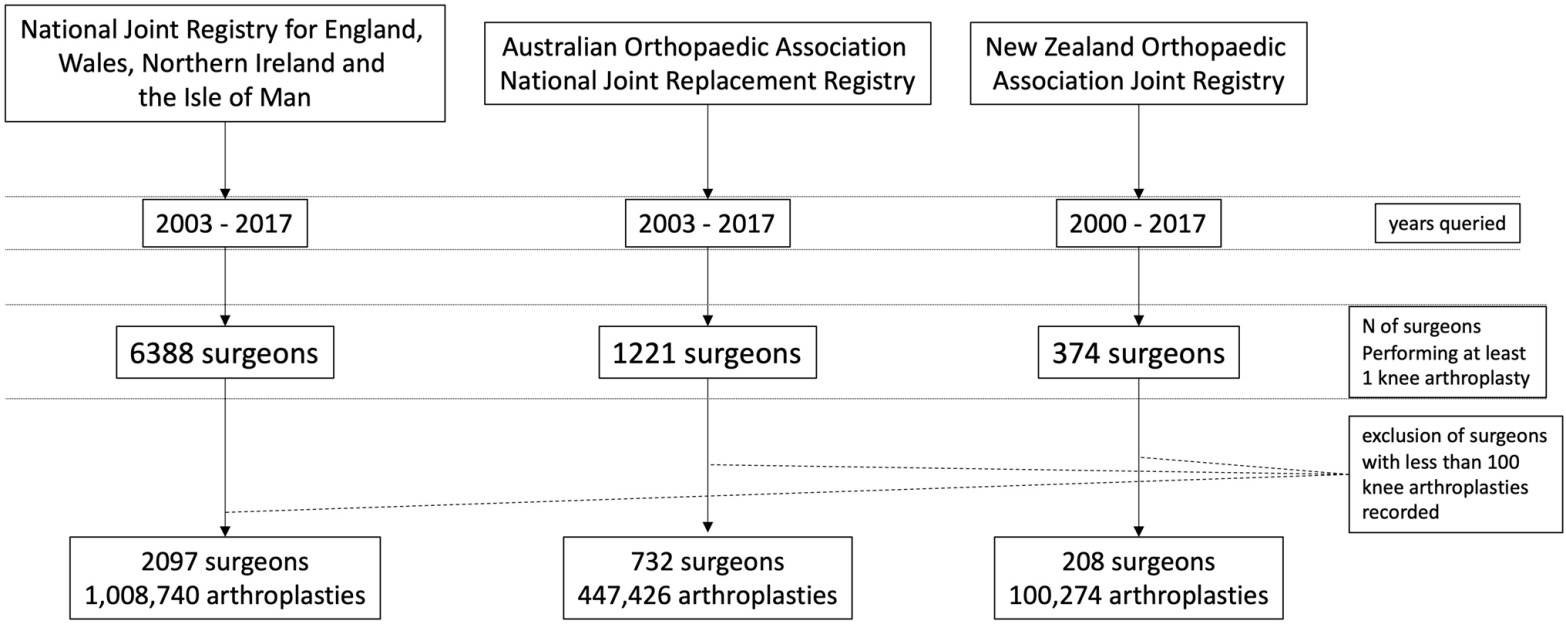
Flow chart of data aggregation and analysis

Within the study period, after data pooling, we identified 1,556,440 knee arthroplasty procedures performed by 3037 surgeons (Fig. 1). Of these, 131,575 were UKA (8.45%), averaging 512 arthroplasty cases per surgeon, median 368 (interquartile range 469). Median procedure number of UKA per year, per surgeon was 0.6 (IQR 0–3.4), whereas the median number of TKA per year, per surgeon was 28 (IQR 15.8–51.5). In both the NZ and AUS registries, the usage of UKA peaked at about 20% occurring around 2003, and has declined since then until 2016, Fig. 2.

**Fig. 2:**
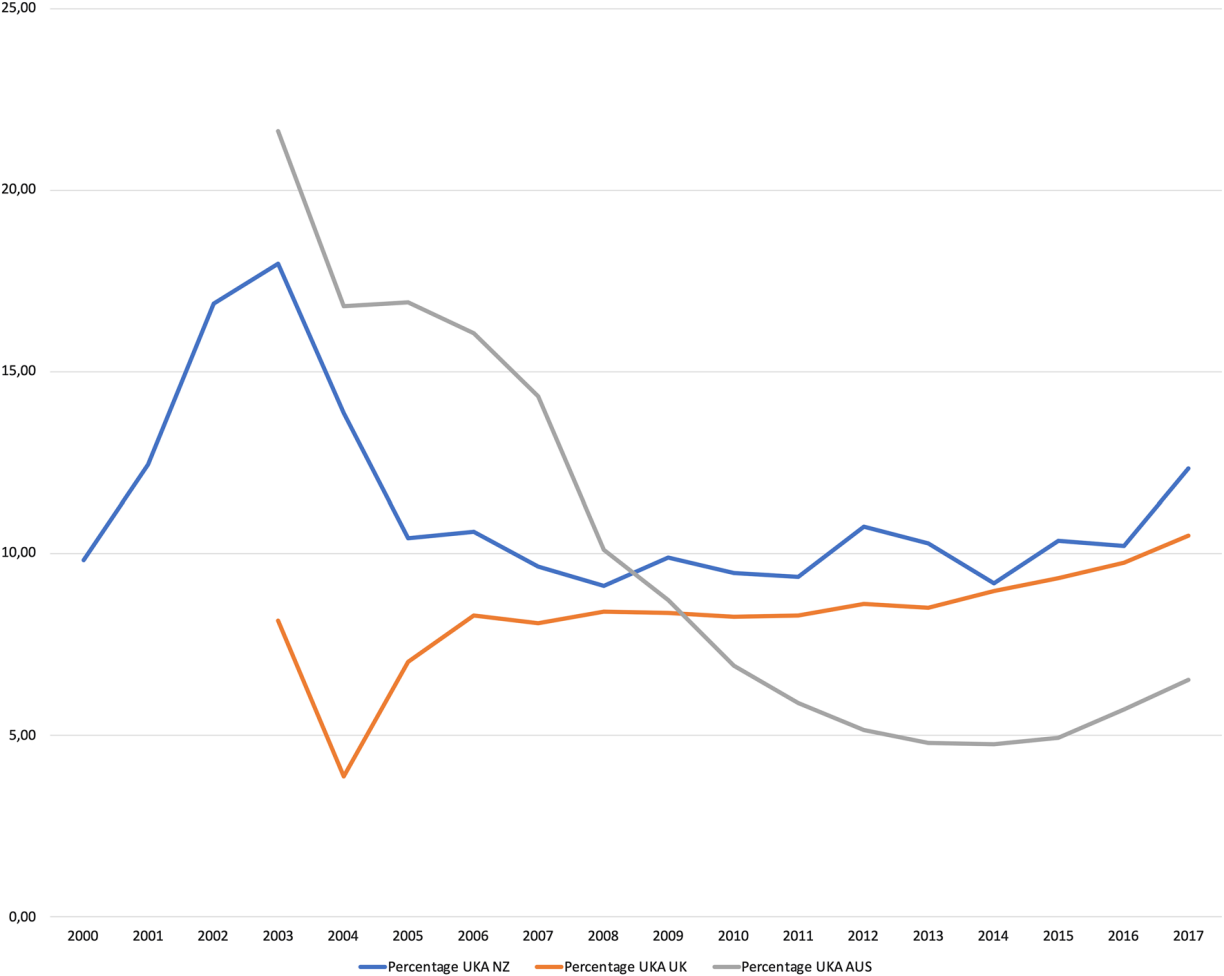
UKA usage over the study period in the studied registries

**Fig. 3:**
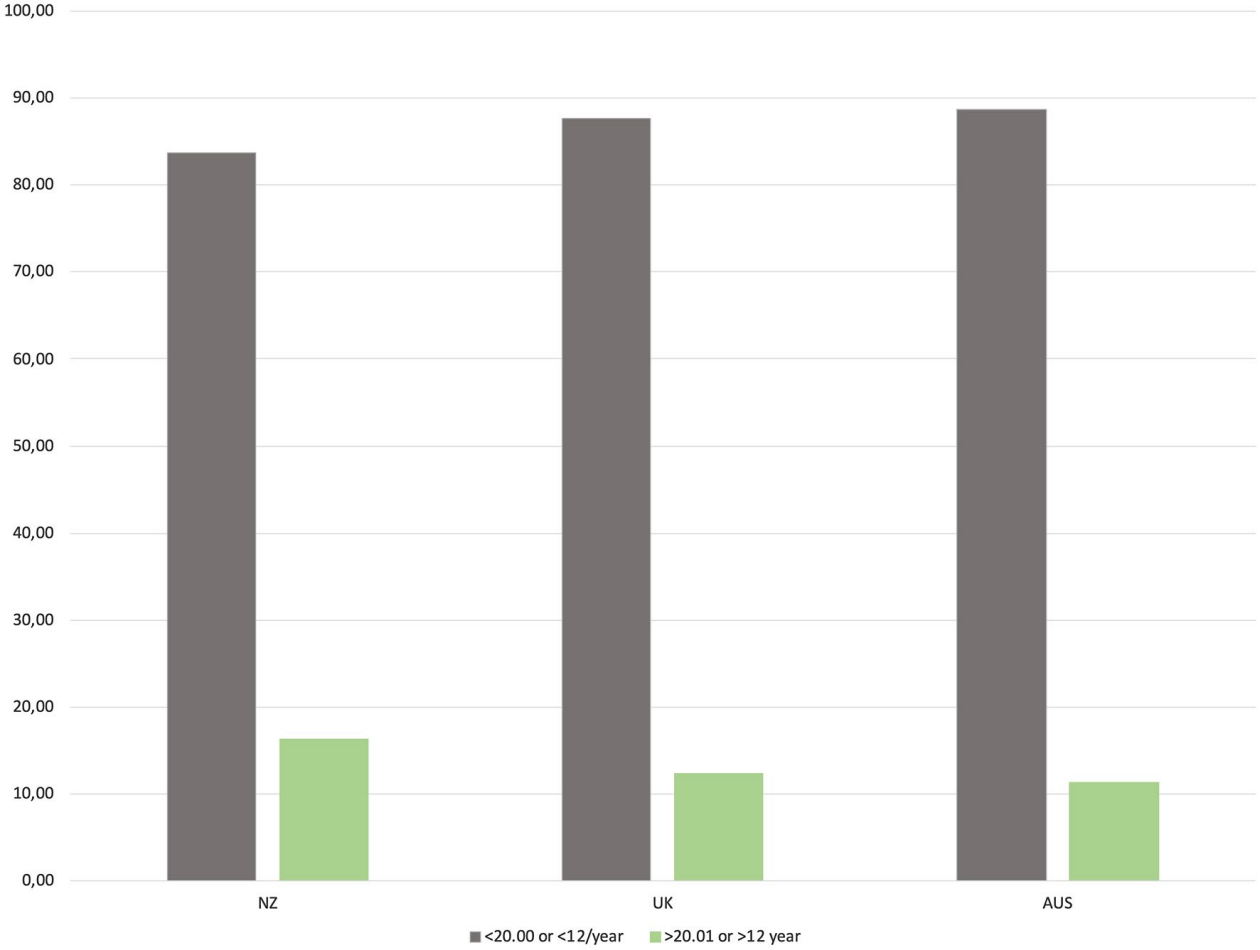
Percentage of surgeons meeting either > 20% UKA or > 12 UKA yearly, per registry

In the study time period, the percentage of knee surgeons performing > 20% UKA yearly was 10.5%. The percentage of knee surgeons performing at least 12 UKA yearly was 7.6%. The percentage of surgeons meeting at least one of the two criteria was 12.1%. It was highest in New Zealand at 16.3%, followed by the UK (12.4%) and Australia (11.3%) (Fig. 3). There was no statistically significant difference in proportion of UKA between the registries (*p* = 0.28).

**Fig. 4:**
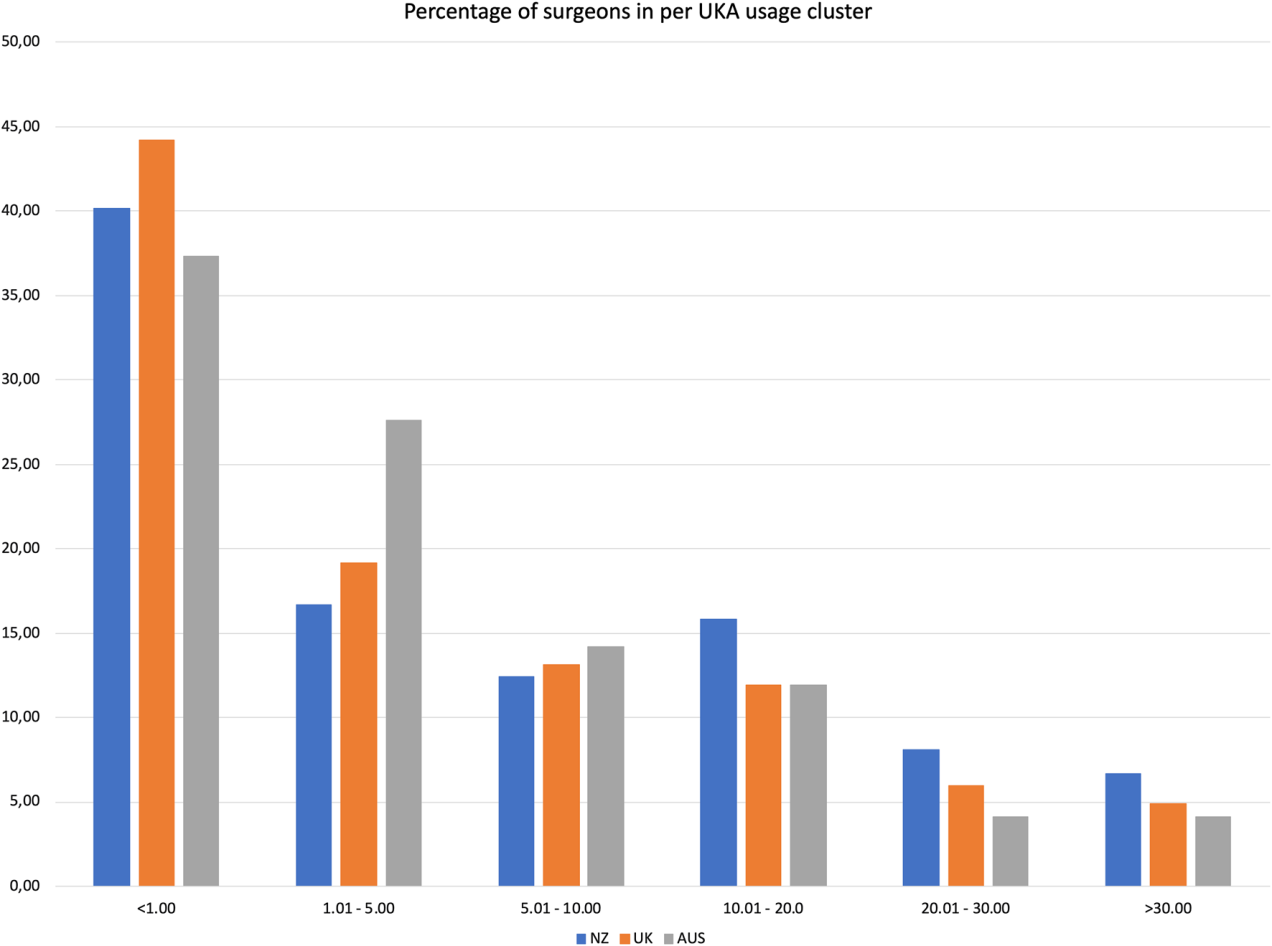
UKA usage, divided into 6 clusters, per registry

More than 50% of knee surgeons in each registry used UKA for less than 5% of their knee arthroplasty procedures, and over 35% of surgeons in each registry used UKA for less than 1% (Fig. 4).

After pooling of data, median surgeon UKA usage was 2.0% (IQR 0–9.1%). In the pooled data, years of activity showed weak positive correlation with increase in UKA usage (*p* < 0.001; *r* = 0.109). There was a weak positive correlation between UKA usage and the overall number of cases performed (*p* < 0.001, *r* = 0.192).

## Discussion

The most important finding of the present study is that a low number of knee surgeons meet the recommended UKA thresholds of > 20% of UKA yearly or > 12 UKA yearly in 3 registries, only 10.5% and 7.6% of surgeons, respectively. Second important finding is the fact that more than 50% of knee surgeons included in this study perform less than 5% of UKA yearly.

Knee surgeon usage of UKA has been shown to impact revision rates [15]. A 20% threshold has been reported by multiple authors [15, 22, 23]. Liddle et al. suggested that ‘optimal’ surgeon UKA usage is in the range of 40–60% [15] whereas Baker et al. reported that a minimum 12 UKA cases per year lowers the revision rate [2]. In fact, one of the first studies reporting a clear relationship between UKA procedure volume and revision rate was a NZ study from 2006, showing that low use surgeons, performing < 1 UKA/year, had an 8% revision rate [8]. A recent meta-analysis of the interaction of knee arthroplasty caseload and UKA usage found that surgeons performing > 30% of UKA can expect results similar to the published long term UKA series [7]. This meta-analysis included 46 studies, with 5 studies not providing information on the proportion of UKA usage. The principle argument from this meta-analysis is that low usage surgeons have a high revision rate, regardless of their annual caseload. The reasons for this are related to the indications for UKA and revision of UKA, not with the surgical technique. The primary indication is anteromedial OA, with bone-on-bone arthritis medially, a normal ACL and MCL, a preserved lateral and patellofemoral compartment without lateral grooving or bone loss [7]. Although a number of previous contraindications such as a high BMI, patellofemoral OA, lower age have been disproven [3, 6, 27, 28], the results of our study suggest that more than half of knee surgeons identify these indications in 5% of cases or less. Even potential differences in patient presentation, demographics and health systems cannot sufficiently account for the discrepancy.

Even in a setting of a knee arthroplasty caseload of 200 procedures per year, < 5% of cases will result in less than the 12 UKA/year. We found a a mean of 45 knee procedures per surgeon per year, therefore 12 UKA would equate to a proportion of 27% usage which only 6% of surgeons achieved. For the majority of cases of knee osteoarthritis, if the above-mentioned criteria are followed, this would potentially result in too few to reach a 20% or 30% threshold. As the main modes of failure of UKA are loosening and disease progression [16] if the indication criteria were to be expanded, the otherwise already comparatively high revision rate of UKA would most likely increase. Furthermore, the proportion of UKA usage per surgeon does not greatly differ between the registries, which demonstrates a consistency in philosophy in the three countries. This suggests that the proposed thresholds may be unrealistic for the average knee surgeon in the present study, and that higher UKA usage may only be achieved by specialist UKA surgeons. The reasons for this discrepancy might be the difference in application of UKA candidacy criteria [1, 30], difference in patient characteristics [5], bias [13, 21] or a combination of these factors.

Usage over the investigated years demonstrate two trends. The first trend is a decrease from 2004 and 2005 in the NZJR and AOANJRR. This decrease may be related to findings of failure rates, published in this time period, of up to 38% as early as 12 months after surgery due to femoral component loosening [18]. Kort et al. reported 89% survival in 2–7 years follow-up and concluded that a UKA is a demanding procedure [12]. Mercier et al. analyzed 43 UKA that had a 10 year survivorship of 74.7% [19]. These authors argue that exclusion of inappropriate patients and surgical errors could improve the 10-year survivorship to 85.7%. There is a lower revision threshold from UKA to TKA, when compared to TKA [10], and some UKA revisions have been deemed avoidable [11]. The type and reasons for revision, can however, differ, ranging from a UKA being revised to a UKA, towards a complex UKA to TKA revision requiring augments [29]. Any revision is a major event for the patient and a result of a primary TKA after such a revision cannot be always expected [14]. The second trend is a small increase in UKA usage after 2015, observed in all three registries. This can possibly be attributed to an increase in robotically assisted UKA [20, 25]. In 2018, 31.8% of all UKA in Australia were robotically assisted [25]. Reports of improved implant positioning [4] and joint line restitution [9] have helped popularize this method [17].

## Limitations

This study has many limitations. The study design was to determine and compare proportions of UKA that surgeons use in the three registries, without investigating the relevant factors that might influence this, apart from surgeon experience. Henceforth, there has been no consideration of the severity of the knee disease, or if this is equivalent in the countries studied. Additionally, the indications and patient assessment of suitability for UKA may vary between surgeons and with registry. We have no access to radiographic data. There has been no comparison of revision rates or other patient outcomes related to the knee surgery undertaken. The threshold of 100 recorded KA cases within the respective registry was chosen as an estimated of an experienced knee surgeon.

## Conclusion

More than 50% of knee surgeons in UK, Australian and New Zealand joint registries perform less than 5% of UKA yearly. The majority of experienced knee surgeons are not meeting the recommended minimum thresholds, which might indicate that the recommended thresholds are not feasible for the vast majority of knee surgeons. The reasons behind this require further research.

## References

[1] Ahmed GO, ELSweify K, Ahmed AF (2020). Usability of the AAOS Appropriate Use Criteria (AUC) for the surgical management of knee osteoarthritis in clinical practice. Knee Surg Sports TraumatolArthrosc.

[2] Baker P, Jameson S, Critchley R, Reed M, Gregg P, Deehan D (2013). Center and surgeon volume influence the revision rate following unicondylar knee replacement: an analysis of 23,400 medial cemented unicondylar knee replacements. J Bone Joint Surg Am.

[3] Beard DJ, Pandit H, Gill HS, Hollinghurst D, Dodd C, a. F, Murray DW,  (2007). The influence of the presence and severity of pre-existing patellofemoral degenerative changes on the outcome of the Oxford medial unicompartmental knee replacement. J Bone Joint Surg Br.

[4] Citak M, Suero EM, Citak M, Dunbar NJ, Branch SH, Conditt MA, Banks SA, Pearle AD (2013). Unicompartmental knee arthroplasty: is robotic technology more accurate than conventional technique?. Knee.

[5] Franklin PD, Miozzari H, Christofilopoulos P, Hoffmeyer P, Ayers DC, Lübbeke A (2017). Important patient characteristics differ prior to total knee arthroplasty and total hip arthroplasty between Switzerland and the United States. BMC MusculoskeletDisord.

[6] Hamilton TW, Pandit HG, Jenkins C, Mellon SJ, Dodd CAF, Murray DW (2017). Evidence-Based Indications for Mobile-Bearing Unicompartmental Knee Arthroplasty in a Consecutive Cohort of Thousand Knees. J Arthroplasty.

[7] Hamilton TW, Rizkalla JM, Kontochristos L, Marks BE, Mellon SJ, Dodd CAF, Pandit HG, Murray DW (2017). The Interaction of Caseload and Usage in Determining Outcomes of Unicompartmental Knee Arthroplasty: A Meta-Analysis. J Arthroplasty.

[8] Hartnett N, Tregonning R, Rothwell A, Hobbs T (2006). The early failure of the oxford phase 3 unicompartmental knee arthroplasty – an audit of revisions. The New Zealand experience. OrthopProc.

[9] Herry Y, Batailler C, Lording T, Servien E, Neyret P, Lustig S (2017). Improved joint-line restitution in unicompartmental knee arthroplasty using a robotic-assisted surgical technique. IntOrthop.

[10] Johnson WB, Engh CA, Parks NL, Hamilton WG, Ho PH, Fricka KB (2020). A lower threshold for revision of aseptic unicompartmental vs total knee arthroplasty. Bone Joint J.

[11] Kennedy JA, Palan J, Mellon SJ, Esler C, Dodd CAF, Pandit HG, Murray DW (2020). Most unicompartmental knee replacement revisions could be avoided: a radiographic evaluation of revised Oxford knees in the National Joint Registry. Knee Surg Sports TraumatolArthrosc.

[12] Kort NP, van Raay JJAM, Cheung J, Jolink C, Deutman R (2007). Analysis of Oxford medial unicompartmental knee replacement using the minimally invasive technique in patients aged 60 and above: an independent prospective series. Knee Surg Sports TraumatolArthrosc.

[13] Labek G, Sekyra K, Pawelka W, Janda W, Stöckl B (2011). Outcome and reproducibility of data concerning the Oxford unicompartmental knee arthroplasty: a structured literature review including arthroplasty registry data. ActaOrthop.

[14] Leta TH, Lygre SHL, Skredderstuen A, Hallan G, Gjertsen J-E, Rokne B, Furnes O (2016). Outcomes of Unicompartmental Knee Arthroplasty After Aseptic Revision to Total Knee Arthroplasty: A Comparative Study of 768 TKAs and 578 UKAs Revised to TKAs from the Norwegian Arthroplasty Register (1994 to 2011). J Bone Joint Surg Am.

[15] Liddle AD, Pandit H, Judge A, Murray DW (2015). Optimal usage of unicompartmental knee arthroplasty: a study of 41,986 cases from the National Joint Registry for England and Wales. Bone Joint J.

[16] van der List JP, Zuiderbaan HA, Pearle AD (2016). Why Do Medial Unicompartmental Knee Arthroplasties Fail Today?. J Arthroplasty.

[17] Lonner JH, Klement MR (2019). Robotic-assisted Medial Unicompartmental Knee Arthroplasty: Options and Outcomes. J Am AcadOrthopSurg.

[18] Mariani EM, Bourne MH, Jackson RT, Jackson ST, Jones P (2007). Early failure of unicompartmental knee arthroplasty. J Arthroplasty.

[19] Mercier N, Wimsey S, Saragaglia D (2010). Long-term clinical results of the Oxford medial unicompartmental knee arthroplasty. IntOrthop.

[20] Mergenthaler G, Batailler C, Lording T, Servien E, Lustig S (2020). Is robotic-assisted unicompartmental knee arthroplasty a safe procedure?.

[21] Murray DW, Liddle AD, Judge A, Pandit H (2017). Bias and unicompartmental knee arthroplasty. Bone Joint J.

[22] Murray DW, Liddle AD, Liddle A, Dodd C, a. F, Pandit H,  (2015). Unicompartmental knee arthroplasty: is the glass half full or half empty?. Bone Joint J.

[23] Murray DW, Parkinson RW (2018). Usage of unicompartmental knee arthroplasty. Bone Joint J.

[24] National Joint Registry for England, Wales, Northern Ireland and the Isle of Man (2019) The National Joint Registry Annual Report 2019

[25] National Joint Replacement Registry AOA (2019) 2019 Hip, Knee & Shoulder Arthroplasty Annual Report

[26] New Zealand Joint Registry NZOA (2019) Twenty year report January 1999 to December 2018

[27] Pandit H, Jenkins C, Gill HS, Smith G, Price AJ, Dodd C (2011). Unnecessary contraindications for mobile-bearing unicompartmental knee replacement. J Bone Joint Surg Br.

[28] Plate JF, Augart MA, Seyler TM, Bracey DN, Hoggard A, Akbar M, Jinnah RH, Poehling GG (2017). Obesity has no effect on outcomes following unicompartmental knee arthroplasty. Knee Surg Sports TraumatolArthrosc.

[29] Thienpont E (2017). Conversion of a unicompartmental knee arthroplasty to a total knee arthroplasty: can we achieve a primary result?. Bone Joint J.

[30] Willis-Owen CA, Brust K, Alsop H, Miraldo M, Cobb JP (2009). Unicondylar knee arthroplasty in the UK National Health Service: an analysis of candidacy, outcome and cost efficacy. Knee.

[31] Wilson HA, Middleton R, Abram SGF, Smith S, Alvand A, Jackson WF, Bottomley N, Hopewell S, Price AJ (2019) Patient relevant outcomes of unicompartmental versus total knee replacement: systematic review and meta-analysis. BMJ 364 10.1136/bmj.l352 PMC6383371 30792179

